# Working among Rags

**Published:** 1859-01

**Authors:** J. Jardine Murray

**Affiliations:** Edinburgh.


### WORKING AMONG RAGS.

By J. JARDINE MURRAY, Esq., Edinburgh.

In many of the inferior streets and alleys of the metropolis, and other towns, may be seen the tawdry stump-doll, or the dirty bunch of parti-coloured ribbons, overhanging the sign-board which indicates that the keeper of that dingy store is ever ready to give "the highest price for all descriptions of rags and bones, etc."

To such places are conveyed the shrived and worn-out garments no longer attractive to old clothes' men, clippings from the workrooms of the tailor and mantua-maker, and—as the police inform us—the semi-rotten shreds which have been scraped up by many a poor wanderer from among our daily consignments to the dust-cart. On account of "value received" their contributions are made over to the rag-collector, who cares not whence they come. His objects are the profitable increase of his tattered heaps and the speedy stuffing of his canvass or hempen bags; that he may shortly have it in his power to make over a ton of goods to some large dealer.

In the more extensive stores, women are employed to overhaul, dust, and sort the rags according to their fabric and quality. Woollen materials are carefully prepared for the London market, to be ground down into "shoddy," and cunningly resuscitated into cloth by being mixed and interwoven with fresh wool. Rags and mixed fabrics are treated in much the same way, or devoted to the manufacture of some coarser kinds of paper.

Linen and cotton rags are sent to the paper-maker. In the process of sorting, silk and worsted pieces, seams, cordings, buttons, etc., are carefully removed, and thus accumulates a specially refuse heap. From being used in the manufacture of prussiate of potash, rags of this description formerly commanded a good price, but for some reason they were of late no longer in demand for this purpose ; and, while apparently inapplicable to other uses, they were not sufficiently valuable as manure to induce farmers to obtain them. Such refuse accumulations therefore increased within the premises of rag-collectors ; and, being in many cases piled in open court, were exposed to the influence of air and moisture and allowed to rot unheeded.

Hence a real nuisance, and hence a very proper outcry !

It cannot be matter of surprise that the police received numerous complaints from the inhabitants of neighbouring houses. For it may readily be supposed that noxious effluvia would be given off from loose heaps in such conditions, and it might even be argued that something like malarious emanations might result. Perhaps, few men would choose to visit the rag-store, and fewer still would wish to reside in its vicinity. The contents may have passed through many phases of an eventful history ; the woollen portion may very possibly lend weight to the coat of many a man who shall wear his vestment in blissful ignorance of the sources whence it is derived ; the linen and cotton portions are doubtless destined to undergo ennobling transformations, which shall make this substance the means of mental communication between man and man ; but the present condition of the whole is really very inattractive.

Who can wonder then that the rag-collectors received little sympathy ; and that, not content with removing the nuisance which had arisen in the manner explained, the Edinburgh police authorities made an attempt to prevent anyone in the rag-trade from having "more than one hundred weight of rags of any one description in any store, or more than one ton in any open court within the police bounds !"

As might have been anticipated, this attempt met with vigorous opposition from the parties concerned. It was asserted that rags were in no respect injurious, that workers among them are extremely healthy, and that the proposed restrictions would be equivalent to a total suppression of the trade. Meetings were held, and a committee was appointed to collect statistics. A printed list of questions was sent to each rag-collector, and another list to each paper-maker. Full answers were in most cases returned; and, as they have been placed at our disposal, we are enabled to present the following abstracts.

We may remark in passing, that in the most important of the above queries, the exclusive use of the word *epidemic* is unfortunate; for, as its derivation (ἐπὶ δῆμος) implies, this word is only applicable to a prevailing disease, or one which attacks a number of people in the same locality at the same time. The word indicated was, doubtless, *contagious* (contingo) or *catching*, a term referrible to such diseases as are communicable from one individual to another and conveyed to the recipient by particles of matter proceeding from the body of the sick, *e.g.*, carried on articles of clothing which have been converted into rags. But, from a careful examination of the answers above referred to, we are led to believe that such distinction was overlooked; and that, had the more correct term been employed, the returns would have been the same.

These statistics, though one-sided, are certainly interesting. That the answers from which they are compiled, were given in perfect good faith, we do not doubt. But if their strict accuracy be admitted, working among rags would be shown to be an occupation not only uninjurious, but positively beneficial to health.

Workers might be expected to suffer—1. From contagion; 2. From the products of decay of the animal and vegetable constituents of the rags—were such decay permitted to occur; 3. From inhaling and swallowing dust.

Let us shortly consider these influences.

1. When we consider whence a large portion of the home-collected rags are derived, it is difficult to see why they should not frequently be means of propagating contagious diseases. Considerable supplies are annually imported into this country from Leghorn, Trieste, and Palermo; and also from Germany through the ports of Bremen and Hamburgh; and it is equally difficult to understand why these should be free from pestiferous influence. It is worthy of remark, that quarantine was formerly enforced on rags imported from abroad, although we believe such precautions are no longer used.

But uniform testimony to the comparative immunity of

RAGS. *Abstract of Statistics from Paper-Makers, 5th September,* 1856.

| Name of Paper Mill. | How many years have you been in the trade? | Workpeople employed during the year. | Quantity of rags of all descriptions consumed annually. | Rags in store at one time on an average in the year. | Do you consider the storage of rags injurious to the health of your workers? | Workpeople seized with epidemic disease through working among rags. | | | Do you know if your workers are predisposed to disease from working among rags? or if any disease is peculiar to workers among rags? |
|---|---|---|---|---|---|---|---|---|---|
| | | | | | | Seized. | Recov. | Died. | |
| | | | Tons. | Tons. | | | | | |
| Leslie paper mills - - | 9 | 100 | 800 | 150 | Not if dry | 0 | 0 | 0 | No |
| Kevock* - - - | 8 | 50 | 572 | 50 | No | 0 | 0 | 0 | No |
| Kinleith - - - | 12 | 100 | 650 | 150 | No | 0 | 0 | 0 | No |
| Bullionfield - - | | | | | | | | | |
| Portobello - - - | 20 | 55 | 450 | 50 | No | 0 | 0 | 0 | No |
| Greenock - - - | 20 | 65 | 550 | 50 | No | 0 | 0 | 0 | No |
| Moffat Mills, Airdrie - | 20 | 100 | 500 | 100 | No | 0 | 0 | 0 | No |
| Stoneywood - - | 70 | 550 | 1800 | 900 | No | 0 | 0 | 0 | No |
| St. Leonard's† - - | 12 | 70 | 400 | 20 | No | 0 | 0 | 0 | No |
| Kates‡ - - - | 15 | 70 | 475 | 125 | No | 0 | 0 | 0 | No |
| Dalbeattie - - - | 29 | 6 | 30 | 4½ | No | 0 | 0 | 0 | No |
| Millbank - - - | 11 | 50 | 300 | 35 | No | 0 | 0 | 0 | No |
| Potton§ - - - | 40 | 185 | 600 | 230 | No | 3 | 3 | 0 | No |
| Balerno Bank - - | 14 | 57 | 512 | 15 | No | 0 | 0 | 0 | No |
| New Battle - - - | 30 | 126 | 1500 | 100 | No | 0 | 0 | 0 | No |
| Strath Mill - - - | 10 | 7 | 25 | 2½ | No | 0 | 0 | 0 | No |
| Muggie Moss‖ - - | 36 | 80 | 1100 | 275 | No | 0 | 0 | 0 | No |
| Vallyfield¶ - - - | 90 | 480 | 1450 | 365 | No | 0 | 0 | 0 | No |
| Chirnside - - - | 66 | 180 | 1200 | 200 | No | 0 | 0 | 0 | No |
| Crook of Dearn - - | 6 | 6 | 60 | 10 | No | 0 | 0 | 0 | No |
| Esk Mills - - - | 35 | 250 | No return | 300 | No | 0 | 0 | 0 | No |
| Cheddar - - - | 17 | 100 | 150 | 20 | No | 0 | 0 | 0 | No |
| Caldercrinx, Airdrie - | 30 | 100 | 750 | 60 | No | 0 | 0 | 0 | No |

* The workpeople are fully as healthy as out-door workers. † The workers are even more healthy than the other residents in the village, who are employed at the carpet-works or in the fields. ‡ The proprietor's dwelling-house immediately adjoins the rag-stores. § Recently, the village of Loanhead was almost decimated by cholera. A large portion of our workers reside in this village; and there was not one death among them, but only two or three slight cases. ‖ The dust from the rags is injurious to the lungs. ¶ Some of those who work long at rag-cutting suffer in old age from shortness of breathing; but among a hundred and forty, who are constantly so occupied in our mill, there are very few such instances.

### *Abstract of Statistics from Rag-Collectors, Edinburgh, September* 1856.

| Places of Business. | How long have you been in trade? | People employed on an average during the year. | Quantity of rags of all descriptions collected in the year on an average. | Workpeople seized with epidemic disease. | | | From your own experience, do you consider rags, when properly packed in bales, injurious to health? |
|---|---|---|---|---|---|---|---|
| | | | | Seized. | Recovered. | Died. | |
| | Years. | | Tons. | | | | |
| 163, West Port - - - | 6 | 3 | 80 | 0 | 0 | 0 | No |
| 177, Cowgate - - - | 5 | 5 | 26 | 0 | 0 | 0 | No |
| 7, Hays Court - - - | 7 | 4 | 160 (incl. bones) | 0 | 0 | 0 | No |
| 8, West Bow - - - | 5 | 2 | 15 | 0 | 0 | 0 | No |
| 6, Low Market Street - - | 12 | 3 | 90 | 0 | 0 | 0 | No |
| 32, Cowgate - - - | 13 | 3 | No return | 0 | 0 | 0 | No |
| 40, Grass Market - - - | 7 | 4 | 15 | 0 | 0 | 0 | No |
| 49, West Port - - - | 20 | 2 | 50 | 0 | 0 | 0 | No |
| 33, George IV Bridge* - - | 30 | 10 | 200 | 1 | 0 | 1 | No |
| 237, Canongate† - - - | 12½ | 3 | 75 | 1 | 1 | 0 | No |
| 57, Tollbooth Wynd, Leith - - | 2½ | 8 | 52 | 0 | 0 | 0 | No |
| Links Pottery, Kirkaldy - - | 45 | 3 | 135 | 0 | 0 | 0 | No |
| Coatfield Lane, Leith - - | 6 | 16 | 300 | 0 | 0 | 0 | No |
| 27, West Preston Street - - | 14 | 1 | 19 | 0 | 0 | 0 | No |
| 5, Tobago Street - - - | 1 | 2 | 8 | 0 | 0 | 0 | No |
| 84, Candlemaker Row‡ - - | 7 | 19 | 375 | 0 | 0 | 0 | No |
| 10, Bristo Place - - - | 3 | 9 | 300 | 0 | 0 | 0 | No |
| 203, Pleasance - - - | No return | 1 | No return | 0 | 0 | 0 | No |
| 54, Blackfriars Wynd - - | 4 | 3 | 50 | 0 | 0 | 0 | No |
| 114, Cowgate - - - | 5 | 1 | 2 | 0 | 0 | 0 | Not if dry |
| 149, Canongate - - - | 8 | 1 | 25 | 0 | 0 | 0 | No |
| 4, Crosscauseway - - - | 25 | 3 | No return | 0 | 0 | 0 | No |
| 60, West Port - - - | 6 | 2 | 100 | 0 | 0 | 0 | No |

* Only one death among our rag-workers during the last ten years. (But from what? old age or epidemic disease?) † This one woman was ill with fever. After a month's absence, she returned to her work. ‡ If the rags are kept dry and properly warehoused, they cannot be injurious.

rag-workers from contagious diseases is not to be lightly set aside.

2. Pernicious influences might doubtless result from decay of the animal and vegetable constituents of rags, But, as the rags would be materially injured by decay, it is seldom or never permitted to occur.

In England, France and Holland, it was formerly the custom to place wet rags in tubs; and after being allowed to ferment for fourteen days they were bruised and hammered into pulp. Great improvements in machinery have now rendered this preparation unnecessary.

As the most refuse rags are again in demand, and as the fibre of all rags is injured by continued exposure to moisture, the stores are invariably kept dry. In paper-mills the rags are now reduced to pulp, not by maceration, but entirely by mechanical means ; and, if any injurious emanations are given off, the chlorine from the bleaching troughs may have a salutary influence.

3. In the preparation of rags it is always necessary to remove great quantities of dust. For this purpose the rags are enclosed in a large wire-cloth cylinder, to the axle of which a number of spokes are fixed transversely. The cylinder is then made to revolve with some velocity. The spokes prevent the rags from remaining in a mass; and the dirt, sand, and dust escape in large quantities through the wire-cloth. That this dust proves in the long run injurious to health, seems not to be disputed.

In many other trades it is known that injury is sustained by those who during their labour necessarily inhale an atmosphere more or less loaded with irritating matter.*

The injurious effects of dust to workers in flax were long ago recorded by Ramazzini :—"Pulvis enim teter ac noxius ex hac materiâ evolat, ut per os fauces, pulmones subiens, operarios ad continuam tussim compellat, ac ad asthmaticam passionem sensim deducat. . . Colore faciei pallido, tussiculos, asthmaticosos ac lippos."†

---

* Among such may be mentioned—millers, maltsters, snuff-makers, flock-dressers, feather-dressers, golf-ball makers, 'shoddy'-grinders, weavers of coverlets, preparers of hair, hatters, 'grounders' of Spanish leather, workers in flax, dressers of hemp, ware-grinders, dressers of japanned goods, masons, colliers, miners of lead (who are injured by working ore in sandstone, but suffer little inconvenience from working ore in limestone), fork-grinders, needle-pointers, file-cutters, those employed in turning and draw-filing cast iron, and the makers of fire-arms who are employed in grinding the barrels of muskets and fowling-pieces.

† RAMAZZINI, De Morbis Artificum, 1717.

Mr. Thackrah has very fully investigated this subject; and, as the dust in flax-mills does not materially differ from that given off during the preparation of rags, we venture to quote from his remarks. "Dressers of flax," he says, "and persons in the dusty rooms of the mills are generally unhealthy. They are subject to indigestion, morning vomiting, chronic inflammation of the bronchial membrane, inflammation of the lungs, and pulmonary consumption. The dust, largely inhaled in respiration, irritates the air-tubes, produces at length organic disease of the bronchial membrane or of the lungs themselves, and often excites the development of tubercles in persons predisposed to consumption.

The first effects, however, do not appear to be direct. The operatives suffer not so much on entering the mill as on leaving it at night. They become particularly susceptible to atmospheric vicissitudes. Besides its application to the air-tubes, dust, we have little doubt, is swallowed with the saliva, and deranges in a greater or less degree the functions of the stomach.

The early stage of the malady which attacks flax-men varies from that of ordinary bronchitis. The cough and difficulty of breathing are not contemporary; one precedes the other, sometimes by months, more frequently by years. The cough is harsh; its invasion is generally confined to the morning and evening."*

We have lately had under medical treatment the foreman of a flax-mill near Lille, in the north of France. This patient suffers from chronic pulmonary catarrh, which he attributes to having worked all his life among flax. The information he has afforded us as to the health of his work-people is in great part confirmatory of Mr. Thackrah's statements.

All the evidence we have been able to collect, certainly goes to prove that rag-collectors suffer from the same affections, only in a less degree. They probably inhale the irritating dust less largely and less continuously than workers in flax do. But the question is only one of quantity and of time.

Various protective appliances have been suggested for their benefit; but, as in analogous cases, such appliances are almost never used. It is evident that none, if possible, should be employed as rag-sorters, who show any tendency to scrofulous disease.

In conclusion, we may remark that the whole inquiry is

---

* The Effects of Arts, Trades, and Professions, on Health and Longevity. By C. Turner Thackrah, Esq. 2nd ed. 1832. pp. 71-2.

evidently beset with fallacies. The consideration of the effects of working among rags may be interesting, and likely to lead to important deductions; but the materials of which we are in possession cannot be considered exhaustive. For, in order to ascertain with precision to what degree any trade is injurious to health, it would be necessary to ascertain the whole number of persons engaged in that trade, the total number of deaths from each disease which occurred among them, and also the results compared with the aggregate mortality of the population in the districts where such trade is carried on. "Possessed of such data upon a sufficiently extensive scale, we might arrive at accurate conclusions respecting the influence of occupation in the production of disease; and having established the aggregate effect of the circumstances connected with the exercise of any particular trade, we might, by a careful study of all such circumstances taken separately, refer each to its proper place in the scale of causes, and determine its positive effect. Researches of this kind, if carefully conducted, could not fail to lead to valuable practical results, by showing what alteration of circumstances might render any particular trade more salubrious."*

Unfortunately, however, the materials for such calculations do not exist.

* A Treatise on Pulmonary Consumption. By Sir James Clark, M.D., F.R.S., 1835, p. 187.

